# Neuromodulation for Chronic Daily Headache

**DOI:** 10.1007/s11916-022-01025-x

**Published:** 2022-02-07

**Authors:** Gianluca Coppola, Delphine Magis, Francesco Casillo, Gabriele Sebastianelli, Chiara Abagnale, Ettore Cioffi, Davide Di Lenola, Cherubino Di Lorenzo, Mariano Serrao

**Affiliations:** 1 Department of Medico-Surgical Sciences and Biotechnologies, Sapienza University of Rome Polo Pontino, Latina, Italy; 2 Headache and Pain Multimodal Treatment Centre (CMTCD), Department of Neurology, Neuromodulation Centre, CHR East Belgium, Verviers, Belgium

**Keywords:** Deep brain stimulation, Sphenopalatine ganglia, Occipital nerve, Vagus nerve, Trigeminal nerve, Transcranial magnetic stimulation, Direct current stimulation

## Abstract

**Purpose of Review:**

We reviewed the literature that explored the use of central and peripheral neuromodulation techniques for chronic daily headache (CDH) treatment.

**Recent Findings:**

Although the more invasive deep brain stimulation (DBS) is effective in chronic cluster headache (CCH), it should be reserved for extremely difficult-to-treat patients. Percutaneous occipital nerve stimulation has shown similar efficacy to DBS and is less risky in both CCH and chronic migraine (CM). Non-invasive transcutaneous vagus nerve stimulation is a promising add-on treatment for CCH but not for CM. Transcutaneous external trigeminal nerve stimulation may be effective in treating CM; however, it has not yet been tested for cluster headache. Transcranial magnetic and electric stimulations have promising preventive effects against CM and CCH.

**Summary:**

Although the precise mode of action of non-invasive neuromodulation techniques remains largely unknown and there is a paucity of controlled trials, they should be preferred to more invasive techniques for treating CDH.

## Introduction

Despite numerous therapeutic advances in recent years, there are several unfulfilled needs in the acute and prophylactic care of primary headaches. Attack therapies are ineffective in one in four patients [1], have numerous side effects and contraindications, and can cause a transformation from episodic to chronic daily headache (CDH) [2]. On the other hand, preventive therapies are ineffective in approximately 50% of patients, have frequent and intolerable side effects, and numerous contraindications [3, 4], leading to poor patient compliance, with more than half of the patients stopping treatment after 2 months. In a recent study of more than 8500 patients suffering from chronic migraine, persistence in oral preventive treatment was 25% at 6 months and 14% at 12 months, with a similar trend even after the second or third prescription [3]. This unsatisfactory picture is further complicated in patients with CDHs, where prophylactic treatment is ineffective in 9 out of 10 patients, many of whom become drug-resistant [3]. Moreover, less than 60% of patients are willing to take any of the drugs available, even if they could benefit from them [5]. Although the situation has improved, it has not yet been completely resolved with the advent of monoclonal antibodies against calcitonin gene–related peptide, the molecule, or its receptor, where the therapeutic response is in line with the ‘old’ prophylactic therapies; however, the adverse events are much less, which means that there is better adherence to treatment [6].

For all these reasons, numerous non-pharmacological approaches that are invasive and risky, such as deep brain stimulation, stimulation of the great occipital nerve, and transcutaneous electrical stimulation, have been attempted during the last decades.

In this review, we will explore studies that have used non-invasive and invasive neuromodulation techniques for the purpose of non-pharmacological treatment of CDHs, such as chronic migraine (CM) and chronic cluster headache (CCH).

## Non-invasive Neuromodulatory Techniques

### Transcranial Magnetic Stimulation

**Fig. 1:**
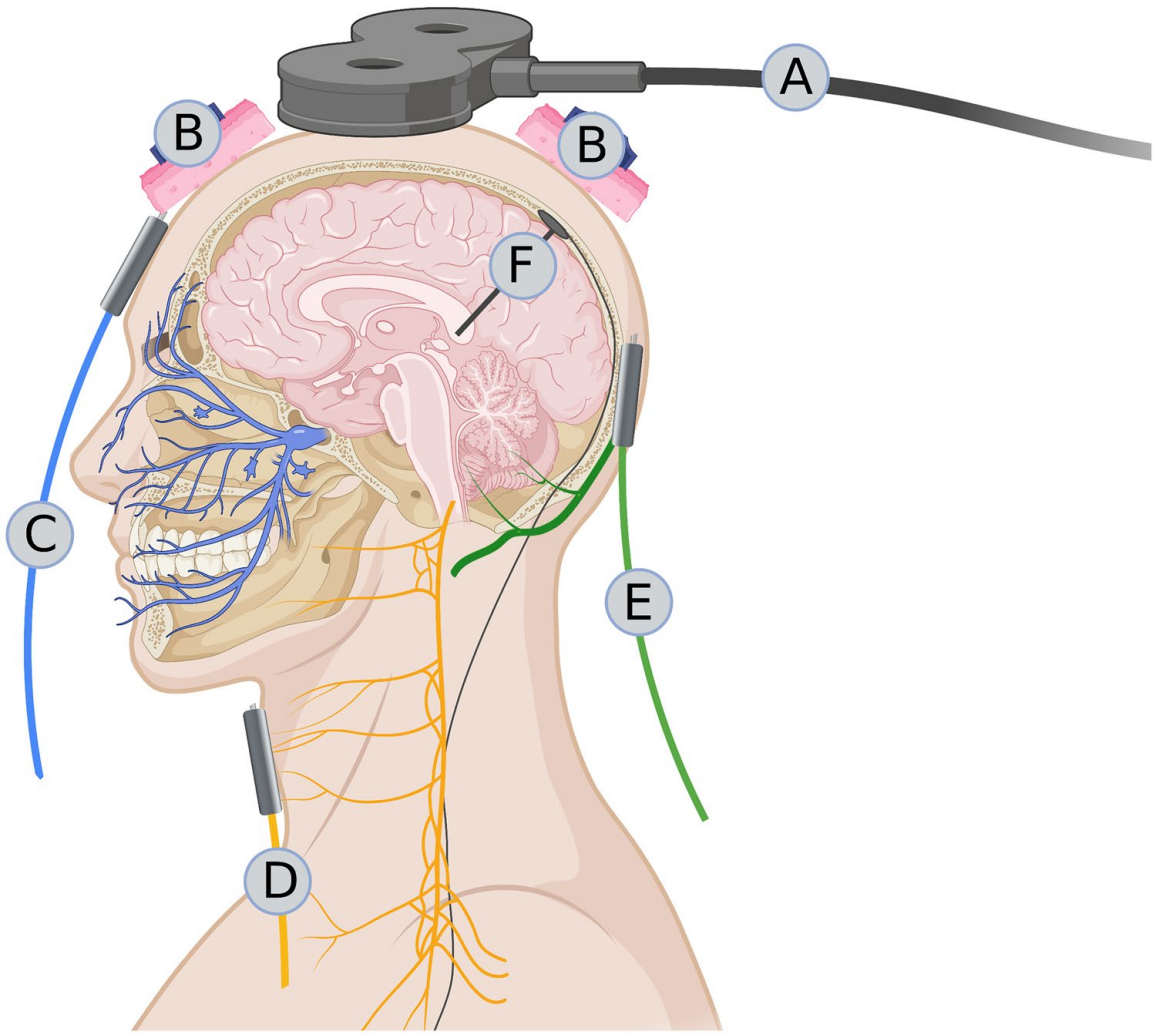
Schematic representation of the sites of possible neuromodulatory intervention in chronic daily headaches. **A** Transcranial magnetic stimulation on the scalp, **B** direct current stimulation on the scalp, **C** transcutaneous stimulation of the supraorbital branch of the trigeminal nerve, **D** transcutaneous stimulation of the vagus nerve at the neck, **E** percutaneous stimulation of the great occipital nerve, and **F** deep brain stimulation of the posteroinferior hypothalamus or ventral tegmentum (Created with BioRender.com)

The rationale for the use of TMS in migraine derives from human studies and studies on animal models, where a single pulse of TMS is able to interrupt the cortical spreading depression, the electrocortical phenomenon at the base of the migraine aura [7]. Human studies have shown how repetitive (r)TMS can bring back within normal limits the altered cortical responsiveness that is frequently detected in migraineurs during the pain-free period [8–10] (Fig. 1).

After three open studies reported the effectiveness of single-pulse (s)TMS in relieving pain, reducing the pain intensity of a single attack [11], sTMS was tested and found to be effective as prophylaxis therapy when used for several days in patients with episodic and CM, with or without medication overuse [12, 13]. In a unique multicentre, single-arm, open-label study, Starling et al. reported the effectiveness of four sTMS pulses delivered twice daily over the occipital area in patients with episodic (90%) and chronic (10%) migraine who were allowed to continue their prophylaxis during the study. They reported a significant reduction in the number of days with headache/month (−2.75) compared to the baseline, a > 50% reduction in headache days in 46% of patients, a reduction in acute medication days and HIT-6 impact questionnaire, and difference from baseline in total headache days of any pain intensity (mild, moderate, or severe) [13].

Repetitive TMS has been tested as a preventive treatment for both episodic and chronic migraine (Table 1). Several open and sham-controlled studies [14–20], but not all [21–23], have shown that high-frequency rTMS delivered to the motor cortex or dorsolateral-prefrontal cortex are both able to reduce the frequency of migraine attacks or monthly headache days. In some of these studies, the scales of anxiety, depression [20, 24], headache-related disability [15, 16, 18, 19], and subjective perception of headache intensity [15–18, 24] also improved after treatment. The limitation of these studies is the inclusion of a heterogeneous group of patients affected by both episodic and chronic forms, often already on prophylaxis and symptomatic drug abuse. In a comparative study, chronic migraine patients treated with high-frequency rTMS obtained a clinical benefit comparable to that obtained with botulinum toxin injection in the first 2 monthly follow-up visits, but not in the third month, so there was a lack of sustained efficacy [18]. We are aware of only one study using high-frequency rTMS to treat, in an open-label fashion, a group of 19 cluster headache (CH) patients. It reported a beneficial effect of rTMS on the intensity of pain, the number of daily attacks, and the percentage of responders up to 15 days after the intervention [25]. Unfortunately, the authors did not specify whether the headache of these patients was episodic or chronic.

**Table 1:** Single-pulse transcranial magnetic stimulation (sTMS) and repetitive transcranial magnetic stimulation (rTMS) studies as prophylactic treatment of migraine

**Authors**	**Participants**	**Study**	**Treatment**	**Results**
Bhola et al. [12]	M = 190: MO or MA = 59, CM = 131 (87 with MOH)	Open-label. Telephone survey	V1	The device was effective at reducing or alleviating their migraine pain in 62% of patients; 59% of patients reported a reduced number of headache days, and 72% reported lower HIT-6 score post-TMS after 12-weeks use
Starling et al. [13]	M = 132	Multicenter, prospective, single arm, open-label, observational study	V1	↓in headache days, in days with acute headache medication, and ↓from baseline of HIT-6 impact questionnaire. The 50% responder rate of 46%. Overall, there were no serious adverse events reported
Brighina et al. [14]	CM = 12: sham (*n* = 6) or rTMS (*n* = 6)	Open-label sham-controlled	HF-rTMS over the left dorsolateral prefrontal cortex	↓ attacks frequency at 2 months with rTMS (53%) than sham (7%); therapeutic gain of 46% ↓ abortive pills and headache index with rTMS, not with sham
Misra et al. [15]	M medically refractory = 51	Open-label study	HF-rTMS over the left frontal cortex	↓ headache frequency at 1 month with rTMS (80.4%); all secondary outcome measures (severity, functional disability, migraine index, and rescue medications) were significantly lower in the rTMS group compared to the sham group
Misra et al. [16]	M = 100 (93 MO, 7 MA, 60 CDH, 28 MOH): sham (*n* = 50) or rTMS (*n* = 50)	Randomised, placebo-controlled double-blind study	HF-rTMS over the left frontal cortex	↓ headache frequency at 1 month with sham (33.3%) than rTMS (78.7%); therapeutic gain of 45.4%; improvement in headache severity in rTMS group (CDH > episodic M) compared to sham All secondary outcome measures (VAS score and functional disability) were significantly lower in the rTMS group compared to the sham group
Rapinesi et al. [24]	CM + MOH = 14	Open-label study	HF-deep-rTMS over the bilateral dorsolateral prefrontal cortex	Compared to the sham group, patients receiving real rTMS showed ↓ of pain intensity, frequency of attacks, analgesic overuse, and depressive symptoms during treatment and one month later
Teo et al. [23]	CM = 9	Randomised, sham-controlled, study	HF-rTMS over the right M1	No significant differences in outcome measures were found between real and sham rTMS
Conforto et al. [22]	CM = 14	Randomised, double-blind, parallel-group, single-centre, clinical trial	HF-rTMS over the left dorsolateral prefrontal cortex	↓ headache days at 2 months with sham (58.1%) than rTMS (15.0%); therapeutic gain of 43.1% ↓ pain intensity at 2 months in both treatment groups; ↓ BDI at 2 months with sham, but not with rTMS; ↓ STAI and MIDAS in both groups
Hodaj et al. [25]	CH = 19	Open-label study	HF-rTMS M1 contralateral to pain	↓ of the intensity of permanent pain, the paroxysmal pain, the daily number of painful attacks, and the percentage of responders from baseline to day 15
Kalita et al. [17]	CM + CTTH = 52	Randomised sham-controlled trial	HF-rTMS over the left frontal region	Both 1-session and 3-sessions or rTMS ↓ frequency of headache, % of patients remitted to an episodic form, duration and severity of headache, and number of abortive medications
Kalita et al. [10]	M = 94	Randomised open-label study	HF-rTMS over the left frontal region	VAS score improved by more than 50% in the 78.6% of patients in the real rTMS group, and in the 34.2% of the patients receiving sham stimulation
Shehata et al. [18]	CM = 14	Open-label, randomised study	HF-rTMS over the left M1	↓ of the headache days and severity, quality of life, and headache disability score in the first 2 months equally in both patients receiving rTMS and botulinum toxin type A. At the month 3 the clinical improvement was maintained by botulinum toxin type A, but not by rTMS
Granato et al. [19]	CM + MOH = 14	Randomised, sham-controlled double-blind study	HF-rTMS over the dorsolateral prefrontal cortex	↓ of mean number of headache days, symptomatic drug intake, headache hours and MIDAS in the two groups without significant differences. Mean number of headache days reduced at T3 by 45,5% and 40% in HF-rTMS and sham groups, respectively
Kumar et al. [20]	CM = 20	Randomised, sham-controlled double-blind study	HF-rTMS over the left M1	↓ in headache frequency in the group receiving real rTMS, but not in the group receiving sham rTMS. Any between groups difference was observed for the variables MIDAS, state and trait STAI, and BDI-II scores

*BDI* Beck Depression Inventory, *CCH* chronic cluster headache, *CDH* chronic daily headache, *CH* chronic headache, *CM* chronic migraine, *DLPFC* dorsolateral prefrontal cortex, *CTTH* chronic tension-type headache, *HF* high-frequency, *M1* primary motor cortex, *M* migraineurs patients, *MA* migraine with aura, *MIDAS* migraine disability assessment, *MO* migraine without aura, *MOH* medication overuse headache, *S1* primary somatosensory cortex, *STAI* State-Trait Anxiety Inventory, *tDCS* transcranial direct current stimulation, *V1* primary visual cortex, *VAS* visual analogue scale

Despite the promising results, further controlled trials, including a larger and more selected population of patients, are needed to confirm the real benefits of this non-pharmacological treatment. These studies will benefit from the technological advances provided by neuronavigation [20] and the development of more specific magnetic stimulation paradigms [26].

### Transcranial Direct Current Stimulation

Unlike TMS, tDCS is better tolerated, more portable, less expensive, and easy to use. It has the same capabilities as TMS to modulate brain activity in the opposite way, depending on the polarity of the direct current. It also offers several additional advantages, such as the ability to influence larger cortical areas, being unfocused, the inability to induce action potentials, and the production of fewer physiological artefacts than TMS.

There is a lot of evidence supporting the use of tDCS in the treatment of migraine prophylaxis and CH. First, tDCS has the ability to normalise cortical hyper-responsiveness that is commonly detected in migraine during the intercritical period [27–29]. Second, tDCS can modulate the functional connectivity of cortico-striatal and thalamo-cortical circuits [30]; the former is involved in the chronicity of migraine and the propensity to overuse symptomatic medication [31], and the latter is involved in the recurrence of migraine attacks, as well as in the generation of the symptoms associated with it [32–34]. Moreover, tDCS can modify the strength of resting-state functional connectivity in cortical networks [35] previously involved in migraine pathophysiology [36].

When applied over the visual area, repeated daily sessions of cathodal tDCS (with the anode over the vertex), which enhances cortical excitability, did change migraine-related clinical variables in two randomised sham-controlled trials, including a mixed group of episodic and CM patients [37, 38].

Four small studies using anodal tDCS over the primary motor cortex reported favourable outcomes in both episodic and chronic migraine patients [39–42]. In one study performed in a mixed group composed of episodic and CM patients, Rahimi et al. [43] obtained a favourable outcome using repetitive cathodal stimulation over either the M1 or the S1 cortex. In a recent study, cathodal stimulation positioned both over V1 (with the anode over the supraorbital region) and over the dorsolateral prefrontal cortex (anode contralaterally) was effective as an add-on treatment during the withdrawal program from medication overuse in a small group of patients with CM [44]. In contrast, in a large multicentre, double-blind, placebo-controlled trial with a 1-year open-label study, Grazzi et al. [45] did not rule out a beneficial clinical effect of anodal or cathodal tDCS over the right M1 in both CM and medication overuse headache patients. Because tDCS applied for 10 continuous days was able to increase the metabolism of the subgenual anterior cingulate cortex [46], an area previously involved in chronic drug-resistant CH (rCCH) response to treatment with implanted occipital nerve stimulation [47], Magis et al. investigated the therapeutic efficacy of tDCS in patients with rCCH [48••]. In this open-label proof-of-concept study, researchers tested the therapeutic response to anodal tDCS over the frontal area in rCCH, arguing that it would activate the functionally interconnected subgenual anterior cingulate cortex. They observed that excitatory tDCS delivered daily for 4 weeks induced a 37% drop in weekly attacks frequency and a 50% responder rate of 43%, which is a promising result, especially considering the difficulty associated with treating such patients [48••].

Overall (Table 2), these promising results using classic tDCS suggest that more refined non-invasive stimulation techniques, such as transcranial alternating current stimulation, can be used to modify the abnormal oscillatory neuronal activity that characterises both CM [34] and CCH [49].

**Table 2:** Transcranial direct current stimulation (tDCS) studies as prophylactic treatment of migraine or cluster headache

**Authors**	**Participants**	**Study**	**Treatment**	**Results**
Dasilva et al. [39]	CM = 13	Randomised sham-controlled trial	Anode over M1	↓ in pain intensity after 4 months with tDCS than sham
Andrade et al. [42]	CM = 13	Pilot, double-blind, placebo-controlled, randomised trial	Anode over M1 or DLPFC	Group under DLPFC stimulation exhibited a better clinical performance compared with groups under M1 and sham stimulations. On intragroup comparison, groups DLPFC and M1 exhibited a greater reduction in headache impact and pain intensity and a higher quality of life after real treatment. No significant change was found in the group under sham stimulation
Magis et al. [48••]	CCH = 31	Pilot trial	Anode over Fz	↓ of attacks frequency, duration, and intensity
Mansour et al. [44]	MOH = 18	Pilot, double blind, sham-controlled, and cross-over design	Cathode over V1 of the headache side, anode over the supraorbital area on the non-headache side For prefrontal tDCS, the anode and cathode were respectively positioned over left and right DLPFC	Both locations: ↓of the total number of migraine days, severe migraine days in the week following the intervention. Occipital tDCS: ↓in the consumption of tablets, clinical effects outlasting the follow-up period of 14 days
Grazzi et al. [45]	MOH or CM = 135	Multicentre, double-blind, placebo-controlled trial with ensuing 1-year open-label study	Anodal, cathodal, or sham tDCS over the right M1	Lack of efficacy of tDCS on the reduction of migraine days and analgesics/months variables
Dalla Volta et al. [128]	CM = 45	Pilot, sham-controlled	Cathodal stimulation unilaterally on the frontal cortex over the cold patch as identified by thermographic examination	↓ in migraine days, headache days, attacks duration, pain intensity, tablets intake
Rahimi et al. [43]	M & CM = 45	Randomised, single-blind, and sham-controlled design	Cathode over the right M1 or S1	tDCS over M1 and S1 ↓ migraine pain frequency, duration, and intensity

*CCH* chronic cluster headache, *CM* chronic migraine, *DLPFC* dorsolateral prefrontal cortex, *M1* primary motor cortex, *M* migraineur patients, *MA* migraine with aura, *MO* migraine without aura, *MOH* medication overuse headache, *S1* primary somatosensory cortex, *tDCS* transcranial direct current stimulation, *V1* primary visual cortex

### Transcutaneous Peripheral Cranial and Extracranial Nerve Stimulation

The following two non-invasive transcutaneous peripheral stimulation devices have been tested for the treatment of chronic headaches: the external trigeminal neurostimulation (eTN, *Cefaly* ®) and the external vagus nerve stimulator (eVN, *gammaCore* ®).

#### External Trigeminal Neurostimulation

Experimental evidence suggests that the mechanism of action of eTN is both peripheral, through segmental “gate control” mechanisms, and central, through suprasegmental mechanisms. The eTN is able to reduce the area under the curve of the blink reflex and to reduce the amplitude of the cortical response to a caloric stimulus sent to the forehead but not to the wrist [50]. This suggests that eTN has a predominantly homotopic action, modulating nociception through a segmental trigeminal-specific mechanism or modulation of the suprasegmental pathway. eTN also seems to induce central effects, such as an increase in reaction time, fatigue, and the critical threshold in the flicker fusion test [51]. Three months of eTN treatment normalised the hypometabolism observed on fluorodeoxyglucose-positron emission tomography [52] and the BOLD hyper signal on functional magnetic resonance imaging [53] of the anterior cingulate gyrus in patients with episodic migraine. In addition, thalamo-cortical somatosensory activity also increased transiently after a single session of eTN stimulation [54].

The clinical efficacy of eTN was initially tested in a randomised double-blind sham-controlled trial (PREMICE study) in which a group of patients with episodic migraine achieved a 50% response rate of 38.2% in the verum group versus 12.1% in the sham group. Patients reported no serious adverse events, and the compliance rate was 61% for the verum group and 54% for the sham group [55]. Later, eTN was tested in an open-label setting in 58 patients with CM, with and without symptomatic drug abuse and with and without continuous headache, who underwent 1–2 daily 20-min sessions for 3 months, achieving an encouraging 50% responder rate of 18.97%. When the authors divided the patient group into those with non-continuous headaches and those with continuous headaches, the former had a response rate of 29.41% and the latter 4.17% [56]. In another study, Vikelis et al. treated a group of episodic or chronic migraineurs with unsuccessful topiramate treatment. Twenty-seven patients in the initial 35 completed the 3-month treatment with eTN, 23 were satisfied with the transcutaneous stimulator, and the mean number of headache days decreased from 8.9 to 6.3/month, similar to the episodic and chronic forms [57•]. A more recent study also showed a non-significant difference in primary and secondary outcome comparisons between episodic (*N* = 60) and chronic (*N* = 23) migraine at 8 and 12 weeks of treatment with eTN [58].

#### External Vagus Nerve Stimulator

The interest in vagus nerve stimulation in the treatment of headaches stems from the evidence of efficacy in the treatment of another accessory brain disorder, epilepsy, when refractory to common treatments. As with eTN, eVN has also aroused interest in headaches due to the miniaturisation process of the devices and the evidence that vagal afferents can be activated transcutaneously [59, 60]. Supporting its use in the treatment of headaches is evidence that eVN can inhibit cortical spreading depression [61, 62], has anti-inflammatory properties, can inhibit trigeminal nociception [63–65], enhance central descending modulation of pain [66], and modulate the activity of the hypothalamus, trigeminal spinal nucleus, pontine nuclei, parahippocampal gyrus, and visual cortex [59]; nonetheless, it inhibits the cranial trigeminal autonomic reflex [67–69]. With the introduction of the GammaCore® device, a portable stimulator of the cervical branch of the vagus nerve, several studies have attempted to prevent both migraine and CH attacks. In the EVENT trial, Silberstein et al. [70] enrolled 30 patients with CM who were self-treated with eVN (120 s × 2 on the right side of the cervical vagus nerve, 5–10 min apart, 3 times a day for 2 months) and 29 patients using a sham device. After 2 months of treatment, the verum device was well-tolerated and safe; however, it brought about no significant improvement over the sham device in terms of both primary and secondary outcomes. During the open-label phase, 16 patients who had received verum continued to self-administer stimulation for a further 6 months and reported a reduction of 3.6 days with headache/month at month 8 of treatment. Eleven patients who switched from sham to verum saw a reduction in 2.5 headache days/month at month 6 of treatment. In another small trial, 26 patients with migraine, 7 of them with chronic migraine, applied eVN bilaterally for 12 s twice daily or sham for 2 months without any significant improvement, except for a reduction in the number of most severe attacks/month [71]. Other authors tested the effects of bilateral stimulation with eVN (120 s twice daily) for 12 weeks in a mixed group of episodic and CM patients who did not respond to at least four therapeutic classes of prophylaxis. Episodic and CM patients equally reported a 50% reduction in the pain intensity of headaches, −5.8 headache days/month and −2.8 migraine attacks/month [72]. In a real-world study, 23 patients with CM were self-treated for 90 s bilaterally, three times a day for three months. Unfortunately, only two patients reported a reduction of at least 30% in the number of headache days per month [73•].

Overall, we can deduce that stimulation with eVN, although possibly beneficial in a subgroup of CM patients in some measures, is well-tolerated and safe, has a low magnitude of effect, and is rarely significant in comparison with sham.

eVN has also been used in the treatment of CH, initially to treat acute attacks in patients with episodic CH and as a preventive treatment for CCH. A controlled study included 92 patients with CCH, 48 of whom received verum and standard of care, and 49 received only the standard of care [74]. At the end of the 4-week randomisation period, those receiving verum had a significantly greater reduction in the number of attacks per week than the control group (with a treatment gain of 3.9 fewer attacks/week) and a higher 50% responder rate (40% vs. 8.3%). In addition, the verum group showed a 57% reduction in the frequency of acute drug use. These beneficial effects observed in the group that initially received verum were also maintained during the open-label extension phase, while only slight improvements were observed in those who initially received only standard of care. The patients did not report any serious adverse events related to treatment.

In a retrospective study, Marin et al. [75] analysed data from 29 CCH patients who received eVN funding from the UK National Health Service. After an observation period of 3–6 months, they found that the frequency of attacks decreased from 26.6 to 9.5 attacks/week, and the same decrease was observed for the mean duration and severity of the attacks and the number of acute medications taken with no adverse events.

In a more recent meta-analysis that analysed a pooled population of 225 CH patients (112 episodic and 113 chronic) of whom 108 had received eVN and 117 sham, the eVN was superior in providing improvement at 15 min of the first treated attack compared to the sham only in the episodic group but not in the chronic group [76].

In summary, in CCH, eVN is a promising add-on preventive treatment; however, its efficacy against acute attacks has not been proven.

## Invasive Neuromodulatory Techniques

### Deep Brain Stimulation

DBS was the first neuromodulatory technique to be proposed for the treatment of drug-resistant CCH. Unlike other non-invasive techniques and stimulation of the great occipital nerve, this technique has not been applied for migraine treatment. The rationale for the use of this invasive technique lies in the neuroimaging evidence of the involvement of the posterior inferior hypothalamus in the initiation and maintenance of a CH attack [77]. It is still debated whether the therapeutic effect observed after months is due to the neuromodulatory effect of areas other than the hypothalamus, but closely connected with it, such as those belonging to the pain neuromatrix [78, 79], the descending cortical pain control system [80], and the midbrain tegmentum [81, 82].

According to recent comprehensive reviews [83, 84], 69 patients with drug-resistant CCH, 3 with short-lasting unilateral neuralgiform headache attacks with conjunctival injection and tearing (SUNCT), 1 with paroxysmal hemicrania, and 1 patient suffering from both CH and SUNCT have been treated with DBS of the posterior-inferior hypothalamus to date in the literature. Of the 73 chronic trigeminal autonomic cephalalgias (TACs), after a mean follow-up of 2.2 years, 31.8% were pain-free and 34.2% had an improvement of at least 50%; therefore, the total treatment success rate was 66% [83]. Only one double-blind randomised controlled trial investigating the efficacy of DBS in patients with CCH is available in the literature [85]. Unfortunately, probably due to the short observation period of one month in this study, the switched-on stimulator did not induce a significant reduction in attacks compared to the switched-off stimulator. The therapeutic efficacy of DBS is long-term. In fact, in another case series, a group of 17 CCH patients was followed up for up to 8.7 years, observing an improvement in 70% of patients [86]. DBS takes weeks to show any effectiveness, cannot treat an ongoing attack [87], does not appear to be effective in those who experience attacks on both sides of the head, and attacks may relapse on each interruption during the first years of stimulation [86]. Three patients with drug-resistant SUNCT [88–90] and one patient with paroxysmal hemicrania [91] also benefited from DBS treatment of the posterior-inferior hypothalamus after at least 1 year of stimulation.

According to a review of stimulation coordinates of DBS studies of the posterior hypothalamus observing the involvement of the ventral tegmental area [81, 82], some authors implanted a DBS device in this region in patients suffering from CCH [92–94] and SUNCT [95, 96] and found a reduction in the frequency, severity, and duration of attacks after a median observation period of 18 months in CCH and 29 months in SUNCT. In parallel, quality of life, mood, anxiety, and novelty-seeking scales improved without changing the cognitive function [92–94].

DBS of both the posterior hypothalamus and the ventral tegmental area is effective in the long term for the treatment of CCH and other TACs, even though the only available sham-controlled study reported contrary findings. Because of its possible serious side effects, this intracranial invasive treatment should only be recommended in cases of failure of the extracranial invasive neurostimulation methods [97].

### Sphenopalatine Ganglion Stimulator

The activation of the parasympathetic system during CH attacks and other TACs and their relief after various procedures acting on the sphenopalatine ganglion (SPG) is well known [98]. For these reasons, a randomised controlled trial tested the efficacy of a microstimulator surgically implanted in the posterior wall of the maxillary bone in the pterygopalatine fossa to stimulate the SPG (Pulsante®). It was initially engineered to treat acute CH attacks by transcutaneously activating the stimulator via a remote controller. In the pathway CH-I trial, 28 patients with CCH completed the experimental period, 68% of whom were responders and 25% of whom responded only to acute treatment, 7% responded to both the acute attack and reducing the attack frequency, and 36% of patients responded only to reducing the attack frequency [99]. At the 1-year follow-up appointment, 45% of patients continued to respond to acute treatment of attacks (23% for very severe attacks), while 35% of the initial 36% continued to respond with a reduction in attack frequency, suggesting that the daily use of the SPG stimulator may have a prophylactic effect on attacks [100]. In a larger cohort of 88 patients with CCH in a 12-month open-label prospective study, 55% of chronic patients were frequent responders, and 74% of chronic patients were able to stop, reduce, or remain off all preventives [101].

To summarise, although the SPG stimulator is only indicated in the treatment of acute attacks, one or two daily 15-min stimulations outside the usual attacks could be used as a preventive treatment.

### Occipital Nerve Stimulator

ONS finds its rationale in the modulation of the trigeminocervical complex [102]. It consists of continuous electrical stimulation of the great occipital nerve through a subcutaneous electrode, which induces paraesthesia in its innervation territory.

Several uncontrolled open-label trials have successfully implanted the ONS in patients with drug-resistant CCH [47, 103–114]. The literature shows that a total of 262 patients with CCH were treated and followed up for an average of 39.1 months. These 262 patients had a clinical improvement of at least 50%, with an overall response rate of 66% (Table 3).

**Table 3:** List of open-label trials implanting percutaneous great occipital nerve stimulator in drug-resistant chronic cluster headache. The mean follow-up period of all patients was weighted by the patient number in individual studies. Taking all trials together, a 50% clinical improvement can be observed in 66% of patients

**Authors**	**Number of patients**	**Follow-up (months)**	**Patients with ≥ 50% improvement**
Magis et al. [47, 103, 107]	15	36.8	11
Burns et al. [108, 109]	14	17.5	5
De Quintana et al. [110]	4	6	4
Mueller et al. [114]	10	12	9
Mueller et al. [104]	24	20	21
Fontaine et al. [111]	13	14.6	10
Strand et al. [112]	3	12	2
Leone et al. [113]	35	72	20
Miller et al. [106]	51	39.17	27
Leplus et al. [105]	93	43.8	64
TOTAL	262	39.1	173 (66%)

ONS was found to be an effective treatment in other chronic headache disorders, such as chronic migraine [104, 115], hemicrania continua [116, 117], paroxysmal hemicrania [118], and SUNCT/short-lasting unilateral neuralgiform headache with autonomic symptoms (SUNA) [118–120].

In an open-label prospective cohort study, Miller et al. identified the presence of pain over the occipital area and severe mood disorders at the time of implantation as strongly associated with poor outcomes in ONS, while a prior response to great occipital nerve block was associated with positive clinical outcomes. Nonetheless, their data showed that patients with SUNCT are better responders than patients with CM [121•].

In a unique international, multicentre, randomised, double-blind, phase 3, electrical dose-controlled clinical trial, 131 patients with drug-resistant CCH underwent 24 weeks of ONS at either 100% (*N* = 65, verum) or 30% (*N* = 66, sham) of the individually determined range between paraesthesia threshold and near discomfort [122••]. At the end of the randomisation phase, both groups achieved a 50% response rate in 44.6% of the cases. At the end of the following open phase (at week 50), the group that received verum saw their response rate increase to 50%, while the sham group continued to have a rate of 44.6%. The authors concluded that although at first glance the similar results obtained with the two dosages of ONS might suggest a placebo effect, the sudden and marked improvement of symptoms after ONS following a highly stable initial observation period of 12 weeks in patients with a clear long history of highly drug-resistant CCH supports a strong therapeutic effect of ONS, even at low dosages.

Overall, despite possible adverse events (empty battery, local infection, lead migration, local pain, neck stiffness, or hardware dysfunction), ONS is safer than DBS and SPG stimulation, and the observed frequency improvement is of a similar order to that of DBS.

## Conclusions

After the initial test of risky, invasive neuromodulation procedures, devices allowing non-invasive riskless neurostimulation are becoming more popular, and patients are more willing to try one of these devices than common drug treatments, including monoclonal antibodies against calcitonin gene-related peptide [123].

In short, DBS of the infero-posterior hypothalamus or ventral tegmental area is effective in CCH (but not without risk); therefore, it should only be reserved for patients who are extremely disabled and extremely difficult to treat. Percutaneous ONS has shown similar efficacy to DBS but with less risk to the patient; it is more effective in SUNCT than other TACs and even CM at a lower intensity. SPG stimulation can abort CH attacks and can also be effective in reducing their frequency. Transcutaneous cervical eVN stimulation can abort episodic but not chronic CH attacks and reduce their frequency. Transcutaneous eTN stimulation is also able to abort migraine attacks and may be effective in treating CM but has not yet been tested in CCH. In favour of using one of these devices is the efficacy rate, often in the range of many drug treatments, and this applies much more to prophylactic therapies than to acute treatment of the attack. In addition, tolerance was generally excellent, with no major treatment-related adverse events. However, their mechanism of action is often elusive, and their cost may discourage their use.

TMS and tDCS may have a preventive effect in both migraine and CCH; however, longer trials are mandatory and require more standardised protocols. We hope that with the advent of new guidelines for clinical trials of neuromodulation devices [124], a higher level of scientific rigor and more solid evidence of the efficacy of this type of non-drug approach will be achieved.

Nevertheless, in the next few years, we will certainly see other devices coming onto the market or being tested not only for episodic headaches but also for CDHs. Examples include transcutaneous extracephalic electrical stimulators [125], caloric vestibular stimulators [126], and percutaneous mastoid electrical stimulators [127].

In conclusion, a better understanding of the mechanisms underlying the recurrence of headache attacks, both migraine and CHs, as well as an improved understanding of the mechanisms that favour the transformation of episodic headache into CDH will contribute to the development of new and more target-specific devices that could relieve pain and its accompanying symptoms.
